# Distinctive Recognition of Flagellin by Human and Mouse Toll-Like Receptor 5

**DOI:** 10.1371/journal.pone.0158894

**Published:** 2016-07-08

**Authors:** Vida Forstnerič, Karolina Ivičak-Kocjan, Ajasja Ljubetič, Roman Jerala, Mojca Benčina

**Affiliations:** 1 Department of Synthetic Biology and Immunology, National Institute of Chemistry, Ljubljana, Slovenia; 2 Centre of Excellence EN-FIST, Ljubljana, Slovenia; Indian Institute of Science, INDIA

## Abstract

Toll-like receptor 5 (TLR5) is a receptor of the innate immune system that recognizes flagellin from certain bacterial species and triggers an inflammatory response. The *Salmonella dublin* flagellin in complex with zebrafish TLR5 has been crystallized previously. In the present study, we extrapolate the structure of this complex using structure-guided mutagenesis to determine the recognition modes of human and mouse TLR5 receptors and demonstrate species-specific differences in flagellin recognition. In general, the recognition mode of the mouse receptor can be said to be more robust in comparison to that of the human receptor. All-atom molecular dynamics simulation showed differences between the two receptors within the primary binding region. Using a functional motility assay, we show that although the highly conserved area of the flagellin analyzed in this study encompasses key structural requirements for flagella formation, a direct correlation between immune recognition and structure on the level of amino acid residues is not observed.

## Introduction

Toll-like receptors (TLRs) are germline-encoded pathogen sensors of the innate immune system that recognize conserved microbial patterns and facilitate host protection against invading pathogens. TLR receptors are highly conserved in evolutionary distant species, and a common mode of recognition and activation is conserved within each TLR type. Nevertheless, differences in species-specific recognition have been observed at the level of the host (receptor) and the pathogen (ligand). In many cases, mice show more robust recognition of microbial pathogen-associated molecular patterns (PAMPs) in comparison to humans, such as in the case of TLR9, where the oligodeoxynucleotides (ODNs) comprising a single CpG activate mouse TLR9 but not human TLR9 [1–3], and in the case of TLR4, where tetra-acetylated lipid IVa activates mouse TLR4 but not human TLR4 [4]. Moreover, human TLR2 but not mouse TLR2 can discriminate between tripalmitoylated and trilauroylated peptides [5]. In the context of TLR5, species-specificity was demonstrated for bacterial flagellins [6] and for TLR5 because the mice and chicken receptor yield more potent responses to most flagellins, and mouse TLR5 (mTLR5) has been shown to be less sensitive to flagellin point mutations [7,8].

TLR5 plays a versatile role in vertebrates’ innate immunity. It is expressed in antigen-presenting cells [9–12] and fibroblasts [13]. In intestinal epithelial cells, the receptor is restricted to the basolateral side [14], and it plays an important role in inflammatory bowel disease, where the barrier against bacterial invasion is disrupted [15]. In lung epithelial cells, TLR5 is expressed on the apical side [16], and it plays a role in damaging lung inflammation in cystic fibrosis [17]. Moreover, flagellin acts as a strong adjuvant, and a number of flagellin-based vaccines for infectious diseases employing flagellin and TLR5 signalling have already entered into clinical trials [18–20].

Flagellins across numerous bacterial species share a common domain structure, in which the conserved D0 and D1 domains comprise the core of the protein that is packed via intersubunit interactions into the flagellar filament, while the D2 and D3 domains, which are highly variable in sequence and length, protrude outwards from the flagellar filament [21,22]. Binding of flagellin to the TLR5 ectodomain (ECD) induces receptor dimerization [23]. Activation of TLR5 with flagellin from β- and γ-proteobacteria such as *Salmonella typhimurium* or *Serratia marcescens* triggers the MyD88-dependent signalling pathway and, subsequently, activates the transcription factor NF-κB, leading to the synthesis of pro-inflammatory cytokines. Three separate regions of flagellin involved in TLR5 activation have been identified. The crystal structure of the N-terminal fragment of zebrafish TLR5-N14_VLR_ in complex with *S*. *dublin* flagellin lacking the D0 domain [24] revealed that the primary binding interface for the α-helices of the D1 domain lies on the lateral side of TLR5, stretching from LRRNT to LRR10 [24] and targeting a conserved segment of the flagellin, which is important for flagellar assembly [22,25]. The heterodimer TLR5:flagellin dimerizes to form a 2:2 complex [23]. The convex side of the second TLR5 (TLR5’ LRR12/13) and the C-terminal αND1b of the primary flagellin form a secondary dimerization interface-α, which is thought to stabilize the tetrameric complex despite its small interface surface. A third region of flagellin identified to have a role in TLR5 activation is the D0 domain, which is required for receptor activation. However, its deletion only slightly impedes the binding of such a truncated flagellin variant to TLR5 [24,26,27].

In the present study, we focus on species-specificity in regard to the host species. We constructed molecular models of human TLR5 (hTLR5) and mTLR5 in complex with the D1 domain of *S*. *typhimurium* flagellin based on the crystal structure of zebrafish TLR5-N14_VLR_ (drTLR5) in complex with *S*. *dublin* flagellin [24]. We compared the activities of flagellin and several flagellin point mutants upon stimulation of hTLR5 or mTLR5 experimentally by measuring cell activation and by performing all-atom molecular simulations. Our results indicate species-specific recognition of flagellin with respect to the receptor species and demonstrate that mTLR5 is generally less susceptible to point mutations within flagellin, thus achieving more robust recognition. Despite a few differences in the structures of the binding interfaces between zebrafish and mTLR5 or hTLR5, flagellin recognition appears to target the same conserved region within the D1 domain, as suggested previously [24,25].

## Materials and Methods

### Cell Cultures and Plasmids

The human embryonic kidney cell line HEK293 was cultured in complete media (DMEM, 1 g/l glucose, 2 mM L-glutamin, 10% heat-inactivated FBS (Gibco)) in 5% CO_2_ at 37°C. Human THP-1 monocytes were cultured in RPMI media with 10% FBS. Mouse embryonic fibroblasts (MEFs) isolated from TLR4-defective C3H/HeJ mice were cultured in DMEM, 4.5 g/l glucose media with 10% FBS, streptomycin (100 μg/ml), and penicillin (100 U/ml). All experimental procedures were carried out in accordance to the law of the Republic Slovenia for Food Safety, Veterinary and Plant Protection. The law body granted the permit to the University of Ljubjana, Biotechnical Faculty, Department of Animal Science (Permit Number: U34401-54/2013/3) and approved this study. The animals from which MEFs were collected were cared for in accordance with the dictates of the National Animal Welfare Law of Slovenia and experiments were supervised by expert for animal welfare.

Plasmids pUNO-hTLR5 and pUNO-mTLR5-HA (InvivoGen) coding for hTLR5 and mTLR5, respectively, and pcDNA3 (Invitrogen) were used. Wild-type *S*. *typhimurium* flagellin (SaTy) and its mutants were cloned into the pET19b expression vector (Novagen) and into the pRP4 plasmid (courtesy of E. Miao, Institute for Systems Biology, Seattle) for motility assays. For site-directed mutagenesis, pET19b-SaTy-encoding flagellin of *S*. *typhimurium* was used [28]. The primers used in this study are described in detail in **S1 Table**.

### Production and Isolation of Bacterial Flagellins

*Escherichia coli* BL21 cells transformed with the pET19b plasmid expressing wild-type or mutant flagellin were cultivated at 37°C in a Luria-Bertani (LB) medium containing 50 μg/ml ampicillin. Overnight cultures where transferred to fresh media, grown to an optical density of ~0.8 at 600 nm, and supplemented with 1mM Isopropyl β-D-thiogalactoside (IPTG). Cells where grown at 25°C for 8 h, harvested and lysed in a buffer solution (10 mM TRIS pH 7.5, 1 mM EDTA, 0.1% DOC) containing a protease inhibitor cocktail (Sigma P8849), followed by sonication (pulse 1s on, 2s off, 10–15 min) and centrifugation (12000 rpm for 30 min). 6xHis-tagged recombinant proteins were purified on Ni-NTA affinity agarose (Qiagen) and dialyzed against demi-water. Protein concentration was determined using the BCA assay (Pierce), and purity was confirmed with SDS-PAGE and immunoblotting using murine anti-His antibodies (Qiagen).

### Bacterial Motility Assays

The impact of flagellin mutations on bacterial motility was tested using an immobile bacterial strain *S*. *typhimurium Fli*C *FljB* ATCC 14028s (courtesy of E. Miao, Institute for Systems Biology, Seattle) transformed with a pRP4 plasmid expressing wild-type or mutant flagellin. The bacterial cells were transformed using electroporation at 2.5 kV, 200 Ω, and 25 μF in 10% glycerol. A single colony of transformed bacteria grown on LB agar plates containing 50 μg/ml ampicillin was selected and transferred to the motility test plates (LB media containing 0.3% agar, 1 mM IPTG, and 50 μg/ml ampicillin). The cultures were incubated overnight in the upright position at room temperature, and they were photographed subsequently. Each plate was inoculated with a single colony of *S*. *typhimurium* expressing mutated flagellin and bacteria expressing wild-type SaTy (or those transformed with an empty vector) as the control. Motility of the strains expressing the mutated flagellin was compared to the motility of the control strain transformed with wild-type SaTy.

### Luciferase Reporter Assay

For dual luciferase assays, HEK293 cells were seeded in 96-well plates (Corning) at a density of 2.2 × 10^4^ cells per well (0.1 ml). On the next day, the cells were transiently transfected with plasmids expressing TLR5, pELAM-1 expressing NF-κB-dependent firefly luciferase as a reporter (50 ng per well) (C. Kirschning, Institute for Medical Microbiology, University of Duisburg-Essen, Essen, Germany), and phRL-TK (5 ng) constitutively expressing Renilla luciferase (Promega) for normalizing the transfection efficiency using the JetPEI transfection reagent (Polyplus Transfection). The total amount of DNA in each transfection was kept constant by adding appropriate amounts of pcDNA3 plasmids. After 24 h, the cells were either lysed or the medium was changed and the cells stimulated with purified recombinant flagellin (5 ng/ml) for a further 18 h before lysis. The cells were lysed in a passive lysis buffer (Promega) and analyzed for reporter genes activities using a dual-luciferase reporter assay measuring NF-κB-dependent firefly luciferase and the constitutively expressed *Renilla* luciferase activity. Each experiment was repeated at least twice in at least three biological replicates. Average relative luciferase activity with standard deviation is given. The unpaired two-tailed Student's t-test was used for statistical analysis of the activation efficiency of different flagellins on hTLR5 and mTLR5 comparing the relative luciferase activities (relative light units, RLU).

### Cytokine Detection in human monocytes and mouse embryonic fibroblasts

Human THP-1 monocytes were seeded in 24-well plates at a cell density of 5 × 10^5^ cells/ml and stimulated with 100 ng/ml of flagellin or mutated variants. Six hours later, the cell supernatants were collected, and the concentration of hTNFα (Affymetrix) was determined. The experiment was repeated at least twice in at least three parallels.

MEFs were seeded at 10^5^ per well in a 24-well-plate format and stimulated with 10 ng/ml of flagellin or mutated variants on the next day. Eighteen hours after stimulation, the cell supernatants were collected and analyzed for mIL-6 concentrations using ELISA (Affymetrix). The experiment was repeated at least twice in at least three biological replicates. Average cytokine level with standard deviation is given. The unpaired two-tailed Student's t-test was used for statistical comparison of cytokine expression levels between treatments with different flagellins.

### Immunoprecipitation assay

Soluble ectodomain hTLR5-Fc (InvivoGen, #fc-htlr5) or mTLR5-Fc (R&D Systems, 7915-TR) (8 μg) was incubated with (50 μl) Protein D DynaBeads (Novex) for 90 min at 25°C with shaking at 1000 rpm. Beads were washed 3 times with wash buffer (1xPBS, 0.02% Tween) and incubated with 10 μg or 20 μg of wild-type flagellin or mutants (R90N-E93R, N438D) for 120 min at 25°C with shaking at 1000 rpm. After incubation, beads were washed 3 times with wash buffer and eluted by boiling for 10 min in mixture of 10 μl elution buffer (1% SDS, 1x PBS) and 30 μl 4x SDS-PAGE loading buffer. After centrifugation at 14.000 rpm for 5 min, proteins in equal volumes were separated by SDS-PAGE and probed by Western blot analysis. The h or mTLR5-Fc were detected using Natural Protein A (HRP) (abcam, ab7456) and His-tagged flagellins were detected using mouse Tetra-HIS Antibody (Qiagen) and goat anti-mouse IgG-HRP (Santa Cruz, sc-2005). Unspecific binding was determined by incubating flagellins with beads without TLR5 and an input control represents flagellins loaded to beads.

### TLR5 and flagellin sequence alignment

Flagellin amino acid sequences were aligned using the ClustalW software program (http://embnet.vital-it.ch/software/ClustalW.html). Sequence alignment included γ-flagellins from *S*. *typhimurium* (SaTy, P06179), *S*. *marcescens* (SeMa, UniProt id. P13713), *S*. *dublin* (SaDu, UniProt id. Q06971), as well as ε-flagellins from *Helicobacter pylori* (HePy, UniProt id. Q59HI7) and *Campylobacter jejuni* (CaJe, UniProt id. P22252) that both evade TLR5 activation. A multiple sequence alignment of the 20 most diverse flagellins by Beatson et al. [22] was also used for analysis of amino acid residue conservation.

### Molecular modelling of TLR5 with flagellin

Structural models of the hTLR5 ECD (aa 21–639) and the mTLR5 ECD (aa 27–641) were generated using MODELLER v9.15 [29]. *dr*TLR5 (PDB code 3V47 [24], chain A) was used as the homology template. The TLR5 homology models and the structure of *S*. *typhimurium* flagellin (PDB code 1IO1 [30]) were aligned with *dr*TLR5 (3V47, chain A) and flagelin (3V47, chain C) of the crystallographically determined complex. This procedure was used instead of docking to ensure the correct orientation of m/hTLR5 and flagellin. The ND1 (aa 56–178, flagellin F1) or CD1 domain (aa 400–450, flagellin F2) of flagellin was used for further simulations and upon alignment the various mutants of *S*. *typhimurium* were created. After homology modeling, side chains were repacked using SCWRL 4.0 [31]. The input files for molecular dynamics (MD) simulation were prepared using VMD [32], and the simulations were executed in the NAMD 2.10 environment [33] using the CHARMM 22 all-atom force field with CMAP correction [34]. The TLR5-flagellin complex was first minimized in vacuum over 1880 steps, solvated with explicit water (box size: 83 × 79 × 104 Å^3^), and equilibrated over 12,000 steps. The production trajectory was run for 30 million steps (60 ns); an integrator step size of 2 fs, Langevin thermostat temperature of 300 K, and Langevin barostat pressure of 1 bar were used. Snapshots were saved every 50 picoseconds.

The free energy of binding was estimated using MM-GBSA in NAMD [35]. Briefly, the energies for trajectories of only flagellin, only TLR, and the TLR–flagellin complex were calculated from the simulations performed using explicit water. The free energy (*G*) of each component was estimated using GBSA (solvent dielectric: 78.5, ion concentration: 0.3 mM, surface tension: 0.005 kcal/mol/Å). The free energy of binding was obtained as follows:

$$\Delta G=\frac{1}{N_{frames}}\sum_{i=1}^{N_{frames}}\left(G(complex)-G(flag)-G(TLR)\right)$$

Only the differences between the mutant and the wild-type with the same TLR are directly comparable, and they were calculated using the following formula:

ΔΔG=ΔG(mutant)−ΔG(wild type)

In total 14 simulations of ~60 ns length were performed (mTLR5-flag, hTLR5-flag, mTLR5-flagR90A, hTLR5-flagR90A, mTLR5-flagR90N, hTLR5-flagR90N, mTLR5-flagE93R, hTLR5-flagE93R, hTLR5-flagE93R-R1, mTLR5-flagR90N_E93R, hTLR5-flagR90N_E93R, hTLR5-flagR90N_E93R-R1, mTLR5-flagN438D, hTLR5-flagN438D). The R1 suffix designates an alternative starting roamer of E93R. SCWRL choose different starting rotamers for the hTLR and mTLR complex for the flagellin mutant E93R. To exclude that differences in the binding energy are based only on a different starting rotamer conformation, simulations of hTLR5-flagE93R-R1 and hTLR5-flagR90N_E93R-R1 were performed in which 93R was in the same starting conformation as in the mouse complex. The obtained energies were averaged over a 1 ns window to obtain 60 ΔΔG points that were presented as violin plots.

MDtraj [36] was used for root mean square deviation (RMSD) calculations (**S1 and S2 Figs**) and for computing dictionary of protein secondary structure (DSSP) plots (**S3 Fig**). ERRAT [37] was used to estimate the validity/quality of the models (**S4 and S5 Figs**). The values were calculated using the on-line webserver at http://services.mbi.ucla.edu/ERRAT/. The DOPE Z-score [38], which measures the likeness of the model to native-like proteins, was calculated every 1 ns of the molecular dynamics simulation (**S6 Fig**).

### Software and statistics

UCSF Chimera 1.6.2 software was used to generate structural figures and determine the distances between atoms (http://www.cgl.ucsf.edu/chimera/ Pettersen et al. [39]). Graphs were prepared using Origin 8.1 (http://www.originlab.com/) and matplotlib (http://matplotlib.org/) [40]. GraphPad Prism 5 (http://www.graphpad.com/) was used for statistical analysis.

## Results

### Mouse TLR5 is less sensitive to mutations in flagellin than human TLR5

**Fig 1:**
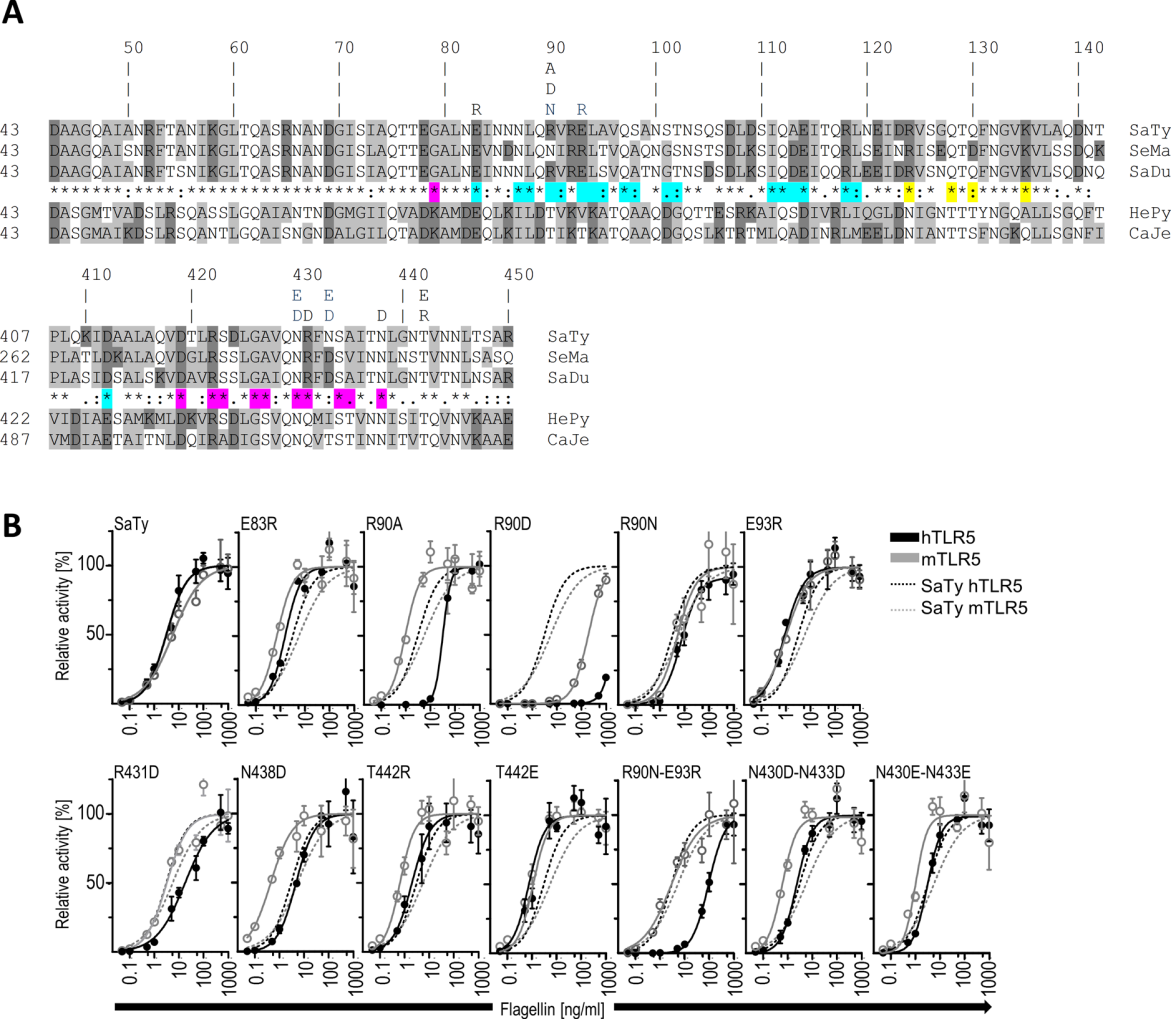
Mapping flagellin residues with presumed effect on receptor species-specific recognition. (**A**) Amino acid sequence alignment of the N- and C- D1 domain of *S*. *typhimurium* SaTy, *S*. *marcescens* SeMa, and *S*. *dublin* SaDu γ-flagellins and N- and C- D1 domain of ε-flagellins of *H*. *pylori* (HePy) and *C. jejuni* (CaJe), which both evades activation of TLR5 (ClustalW). Residues selected for point mutations are shown above the alignment. Hydrophobic aa are represented in light grey and charged aa in dark grey. Amino acids corresponding to *S*. *dublin* residues that form the primary binding interfaces A and B are highlighted in magenta and cyan, respectively, and aa of the secondary dimerization interface are highlighted in yellow [24]. (**B**) Responses to mutated flagellin differ between hTLR5 and mTLR5. For dose-response curves, hTLR5^HA^- or mTLR5^HA^-transfected HEK293 cells were stimulated with up to 1 μg/ml of purified recombinant SaTy or mutants, and luciferase activities were measured. (*Data are representative of two independent experiments*. *Points represent mean of 4 biological replicates ± s*.*d*.) Shown here is the percent fold induction of NF-κB activity relative to stimulation with the maximal concentration of wild-type SaTy (1μg/ml), with the exception of R90D, in which case saturation was not achieved. The response to the mutants (solid lines) is superimposed on the response to wt flagellin (dashed lines).

Differential recognition of flagellins from different bacterial strains has already been demonstrated for the mTLR5 and the hTLR5 receptors [7]. To determine which amino acids within the primary binding interface of flagellin contribute to this species-specific recognition, we selected amino acid residues for point mutations based on their contributions to the binding interface according to the crystal structure. The primary binding site of *S*. *dublin* flagellin (SaDu) to *dr*TLR5 is restricted to the flagellin subdomains αND1 and αCD1, and the ascending lateral interface of *dr*TLR5 LRRNT to LRR10 with approximately 1320 Å^2^ of buried accessible surface area [24]. This region of flagellin is highly conserved because it is packed through intersubunit connections within the flagellar filament. Therefore, an additional consideration for point mutation targets was the conservation of amino acid residues and their contribution to intersubunit connections within the filament. Residues E83, N430, N438, and T442, which are conserved among flagellins, and additional residues R90, E93, R431, and N433, all of which face neighboring flagellin molecules in the flagellar filament, were selected (**Fig 1A**). The charges of the amino acid residues were reversed, and polar amino acid residues were replaced with charged amino acid residues. Mutations R90N, E93R, N433, as well as a double mutant R90N-E93R, were changed to the counterpart residues of SeMa, which has been reported to differentially activate h- and mTLR5 [7]. The substitution of N433 to D was expected to have a minor impact on TLR5 activation and bacterial motility since it is a naturally occurring mutation of SaTy associated with the curly morphology of filament [21]. Residue N430 is also encompassed in the primary binding interface and is positioned just above N433 facing in the same direction in *S*. *typhimurium* flagellin. To intensify the effect of mutations in this region, double mutants N430D-N433D and N430E-N433E where generated. All flagellin variants were purified and analyzed. HEK293 cells transfected transiently with hTLR5 or mTLR5 were stimulated with the mutated flagellin variants, and the NF-κB activation level was measured. The activation of h- and mTLR5 by each flagellin point mutation is shown as a dose response curve relative to stimulation with wild-type *S*. *typhimurium* flagellin (SaTy). The response to each mutant (solid lines) is superimposed on the response to wt flagellin (dashed lines) (**Fig 1B**). The results of the point mutations could be grouped into three classes: those with no effect, those that abrogated TLR5 recognition, and those that reduced only hTLR5 activation but not mTLR5 activation. Mutations E83R, R90N, E93R, T422R, and T422E had no effect on the flagellin’s efficiency to activate TLR5. The charge-reversed variant R90D was the only mutation that significantly reduced the ability of flagellin to stimulate both mTLR5 and hTLR5 (**Fig 1B and Table 1**). Point mutants R90A, R431D, and N438D, of which the corresponding amino acid residues of *S*. *dublin* form the primary binding interface with *dr*TLR5 [24], exerted an effect on activation of hTLR5 but had a minimum or no effect on the activation of mTLR5 (**Fig 1B**). Interestingly, the E93R or R90N mutants alone had no effect on the ability of flagellin to activate h- or mTLR5, whereas a combination of both mutations impaired the activation potential of hTLR5 but had no influence on mTLR5 activation. Double mutants N430-N433 to negative E or D had a minor impact on activation efficiency, but they were still able to activate mTLR5 to a higher degree than hTLR5, even increasing the activity of mTLR5 with respect to wt flagellin.

**Table 1:** Comparison of flagellin functionality and its ability to activate hTLR5 or mTLR5 in SaTy and mutant variants. Activity was determined for mTLR5^HA^- or hTLR5^HA^-transfected HEK293 cells stimulated with 5 ng/ml wild-type flagellin and mutants.

Mutants	Motility		Activity			
	[%]		hTLR5 [%]		mTLR5 [%]	
SaTy	100	+++	100	+++	100	+++
E83R	72	++	112	+++	105	+++
R90A	80	++	0	-	77	++
R90D	2	-	0	-	13	-
R90N	22	+	50	++	77	++
E93R	23	+	120	+++	115	+++
R431D	0	-	43	+	82	++
N438D	75	++	34	+	112	+++
T442R	37	+	82	+++	104	+++
T442E	68	++	89	+++	100	+++
R90N-E93R	59	++	4	-	86	++
N430D-N433D	0	-	98	+++	117	+++
N430E-N433E	14	-	85	+++	121	+++

These results confirm the roles that several amino acid residues within the primary binding site of flagellin play in the species-specific differences between hTLR5 and mTLR5 recognition. The mouse receptor was less sensitive to mutations in all cases in which a difference was observed. This indicates either altogether more robust recognition of flagellin by mTLR5, possibly the stronger homotypic inter-receptor interactions, or a slightly different mode of recognition with respect to the region of flagellin recognized by the mouse receptor.

### Flagellin mutants differentially activate human or mouse cells

**Fig 2:**
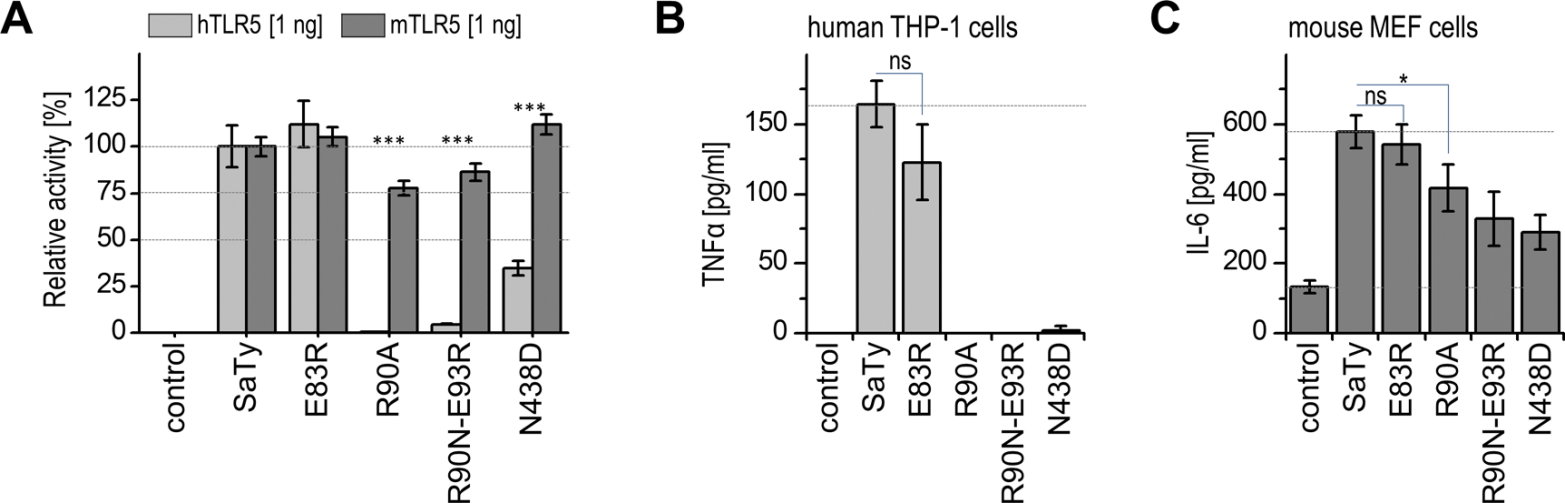
Flagellins with mutations in ND1 and CD1 domains differentially activate human or mouse cells. (**A**) HEK293 cells transfected with a plasmid expressing hTLR5^HA^ or mTLR5^HA^ were stimulated with 5 ng/ml of purified recombinant SaTy or mutant variants and luciferase activities were measured and relative luciferase activities (RLU) were calculated using the *Renilla* luciferase as transfection control. (*Data are representative of 3 independent experiments*. *Bars represent mean of 4 biological replicates ± s*.*d*.; ***p > 0.*005*, *two-tailed unpaired Student’s t-test*). Relative NF-κB activities compared to wild-type flagellin are shown. (**B**) Human monocytes THP-1 and (**C**) MEFs were stimulated with 100 or 10 ng/ml of purified recombinant SaTy or mutants, respectively, and cytokine production was measured (*Data are representative of 2 independent experiments*. *Bars represent mean of 3 biological replicates ± s*.*d*.; ***p > 0.*005*, *p > 0.*1*, *ns*: *p > 0*.*1*, *two-tailed unpaired Student’s t-test*).

To further confirm the relevance of the species-specific recognition of flagellin point mutations between human and mouse receptors, human THP-1 monocytes or MEFs, both expressing endogenous levels of their respective receptors, were tested for cytokine production in response to stimulation with flagellin or mutated variants that elicited the most profound differences in activation. mTLR5 was responsive to flagellin mutants R90A, N438D, and the double mutant R90N-E93R, whereas hTLR5 signalling was abrogated by these point mutations in HEK293 cells (**Fig 2A**). Human THP-1 monocytes or MEFs were, therefore, stimulated using SaTy or the mutated variants, respectively, and synthesis of human TNFα and mouse IL-6 were measured, respectively. The human monocytes were not activated with flagellin mutants R90A, N438D, and the double mutant R90N-E93R (**Fig 2B**), as determined for hTLR5-positive HEK293 cells (**Fig 2A**). In contrast, MEFs were activated upon stimulation with all mutated variants, although some differences in responsiveness can be noticed between the overexpression system in HEK293 cells and primary cells. Specifically, double mutant R90N-E93R and mutant N438D seem to have a greater effect on MEF responsiveness (**Fig 2C**). Taken together, these results confirm the lower sensitivity of mTLR5 to amino acid residue variations in the primary binding site than that of hTLR5.

### Mutations within ND1 and CD1 domains of flagellin affect bacterial motility

**Fig 3:**
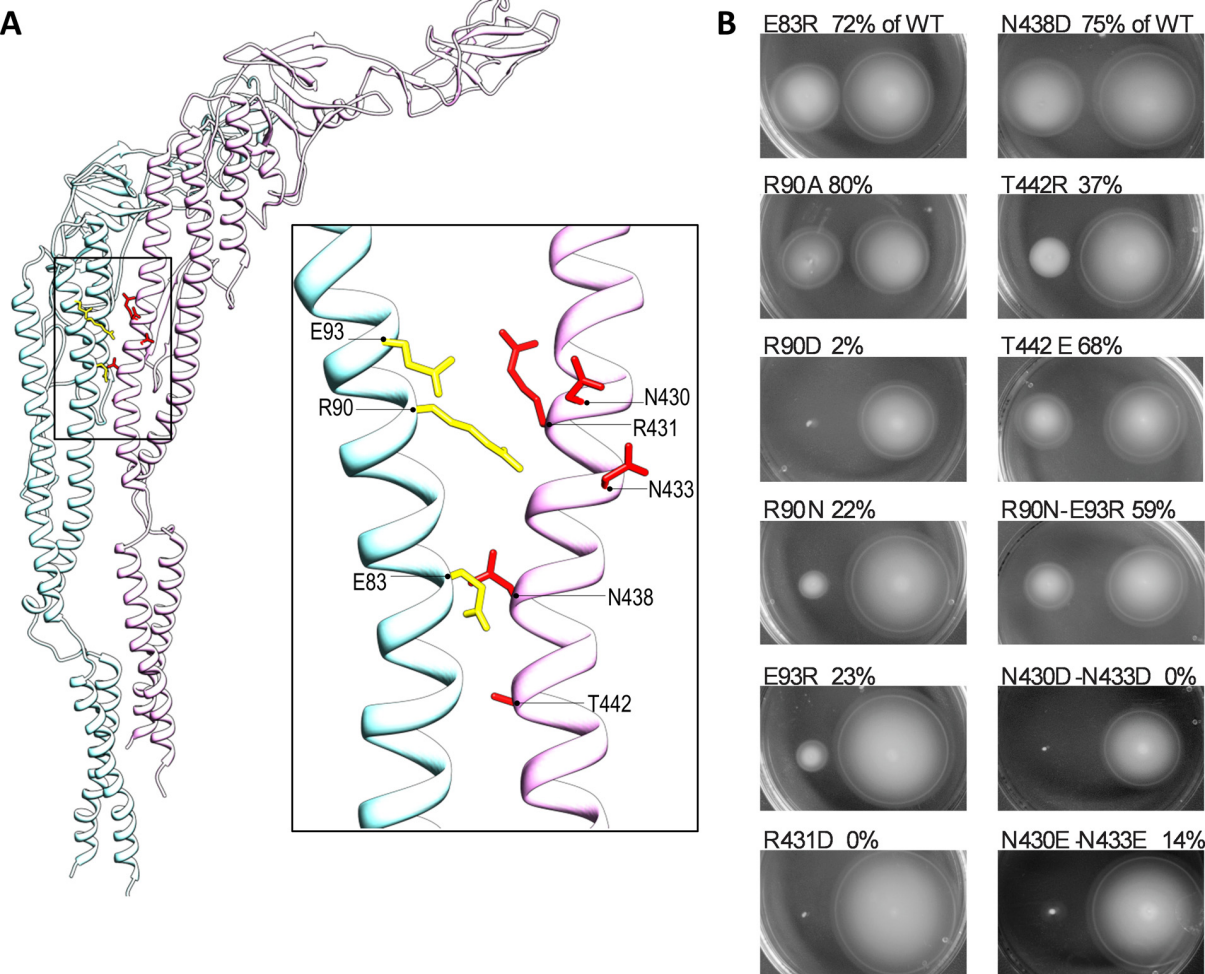
Mutations in D1 domain of SaTy flagellin affect motility of flagellin-deficient bacterial strain *S*. *typhimurium FliC FljB*. (**A**) Amino acid residues subjected to mutagenesis are shown on two adjacent flagellin monomers positioned as in the flagellar filament. Yellow and red colors represent the residues selected for point mutation. (**B**) Motilities of flagellin-deficient bacteria transformed with plasmids encoding mutant or wild-type flagellin were tested on low-density agar plates. Each mutant was inoculated with a wild-type control on the same agar plate (right side). The percentage of mutant versus wild-type diameters is shown. (*Data are representative of 3 independent experiments*.)

Monomers of flagellin self-assemble through intermolecular contacts between the conserved D1 and D0 domains into flagellar filaments that enable bacterial motility [21,30]. The conserved amino acid residues within these regions represent an appropriate conserved target for the innate immune receptor TLR5 because structural requirements restrict extensive modifications, which would allow immune evasion, in this area of flagellin. To assess the impact of the changes in the amino acid residues tested for receptor activation on the functionality of bacterial flagella, mutant flagellins were transformed into a flagellin-deficient strain of *S*. *typhimurium* and tested for their effects on bacterial motility. The positions of specific amino acid residues with regard to their positioning in the flagellar filament between two adjacent monomers are shown in **Fig 3A**. All tested mutants influenced bacterial motility. Mutation R90D and mutations within the C-terminal D1 domain including R431D, N430D-N433D, and N430E-N433E rendered the bacteria completely immobile. Mutations R90N, E93R, and T422R reduced motility by over 50% (**Fig 3B; Table 1**). A strong correlation between activation potential and influence on motility was observed only for the mutant R90D, which rendered the bacteria completely immobile and was unable to stimulate the human or mouse receptor. Double mutants N430D-N433D and N430E-N433E, too, rendered bacteria immobile, but they retained TLR5 activation potential. Within this study, all flagellin variants that were functional for bacterial motility were also detected by TLR5, at least in the case of the mouse orthologue (**Table 1**). This suggests that all conserved amino acid residues tested in this study are under structural restraint as modifications strongly influence flagellar filament formation and result in non-motile or reduced-motility bacteria.

### Comparison of primary binding sites of hTLR5 and mTLR5

Differences were observed upon activation of the hTLR5 or mTLR5 receptor with flagellin containing point mutations in the primary binding site. To evaluate these differences, molecular models of the critical region of the mTLR5 and the hTLR5 complexes SaTy were constructed based on the crystal structure of *dr*TLR5 with *S*. *dublin* flagellin (SaDu) [24]. Due to the high similarity between the sequences of hTLR5 and mTLR5, the homology models of hTLR5 and mTLR5 were highly similar. Because the homology models by themselves cannot explain the observed differences in the activation of hTLR5 and mTLR5, we performed an all-atom molecular dynamics simulation to probe potential differences in binding and to verify our models.

**Fig 4:**
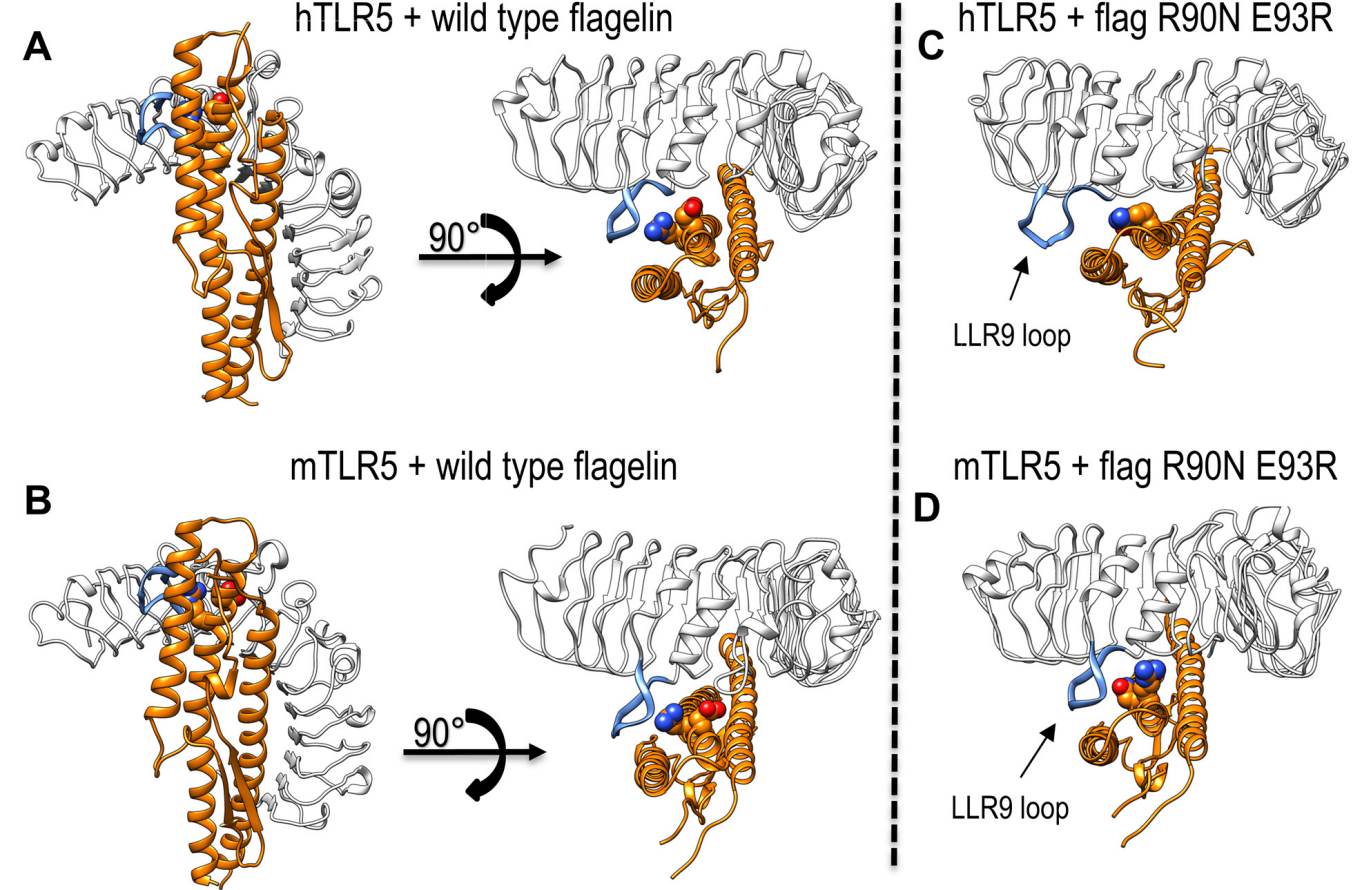
Models of hTLR5 and mTLR5 in complex with wild-type and mutant flagellin. Flagellin is shown in orange, TLR5 in white. The LLR9 loop of TLR5 is shown as a thick blue band. Positions 90 and 93 in flagellin are represented using Van der Waals surfaces. (**A**) Top and side views of hTLR5 with wild-type SaTy flagellin. (**B**) Top and side views of mTLR5 with wild-type SaTy flagellin. (**C**) Top view of hTLR5 with flagellin mutant R90N E93R. (**D**) Top view of mTLR5 with flagellin mutant R90N E93R.

**Fig 5:**
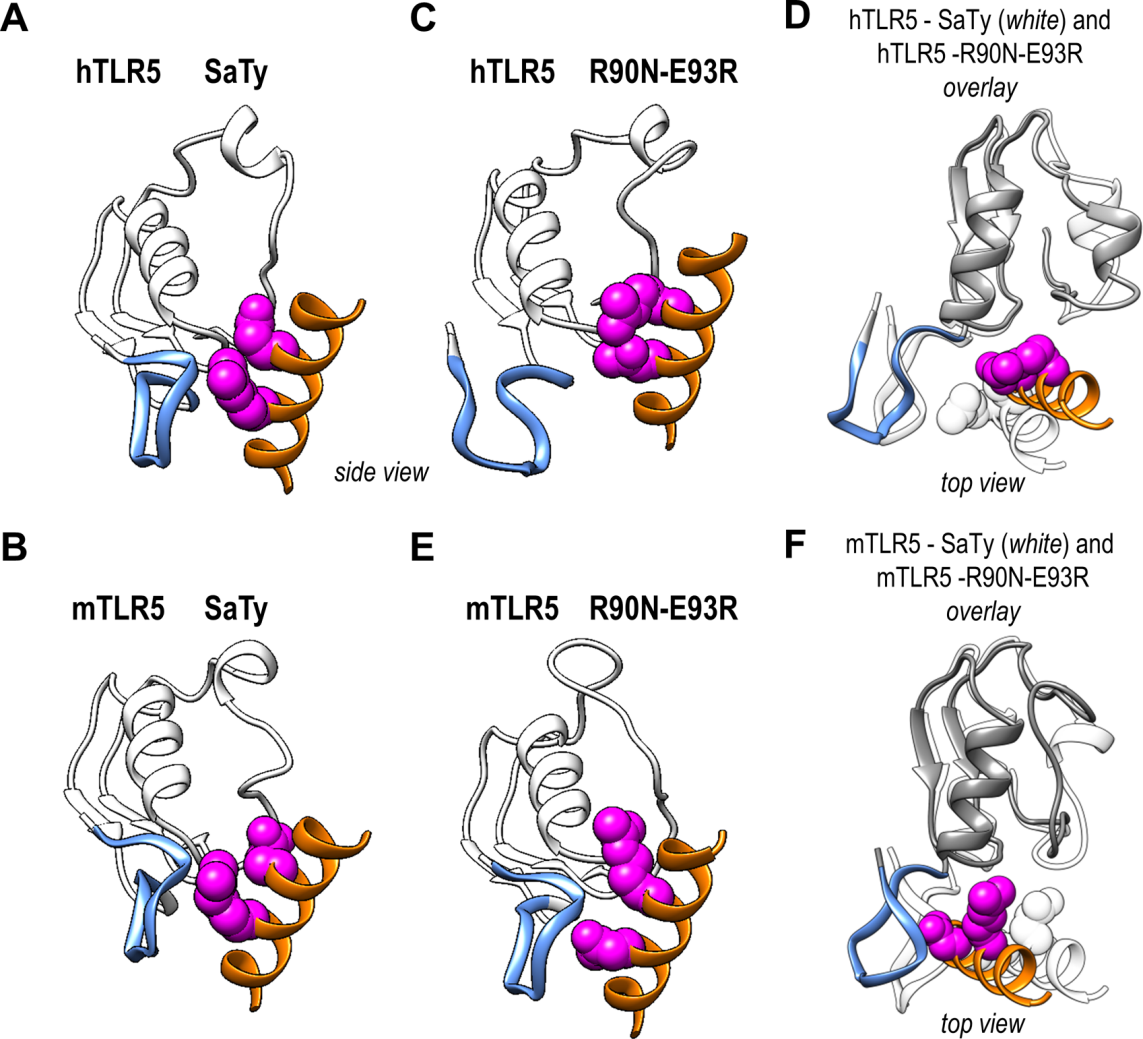
Structural details of binding between wild-type and mutant flagellin. Flagellin is shown in orange, TLR5 in white. The LLR9 loop of TLR5 is shown as a thick blue band. Positions 90 and 93 in flagellin are represented using Van der Waals surfaces. (**A-F**) Zoomed in view of the interaction surface of TLR5 with wt or mutant flagellin (side and top views). The right panels (**D** and **F**) show an overlay of the TLR5 interaction with wt flagellin (white) and mutant flagellin (orange).

**Fig 6:**
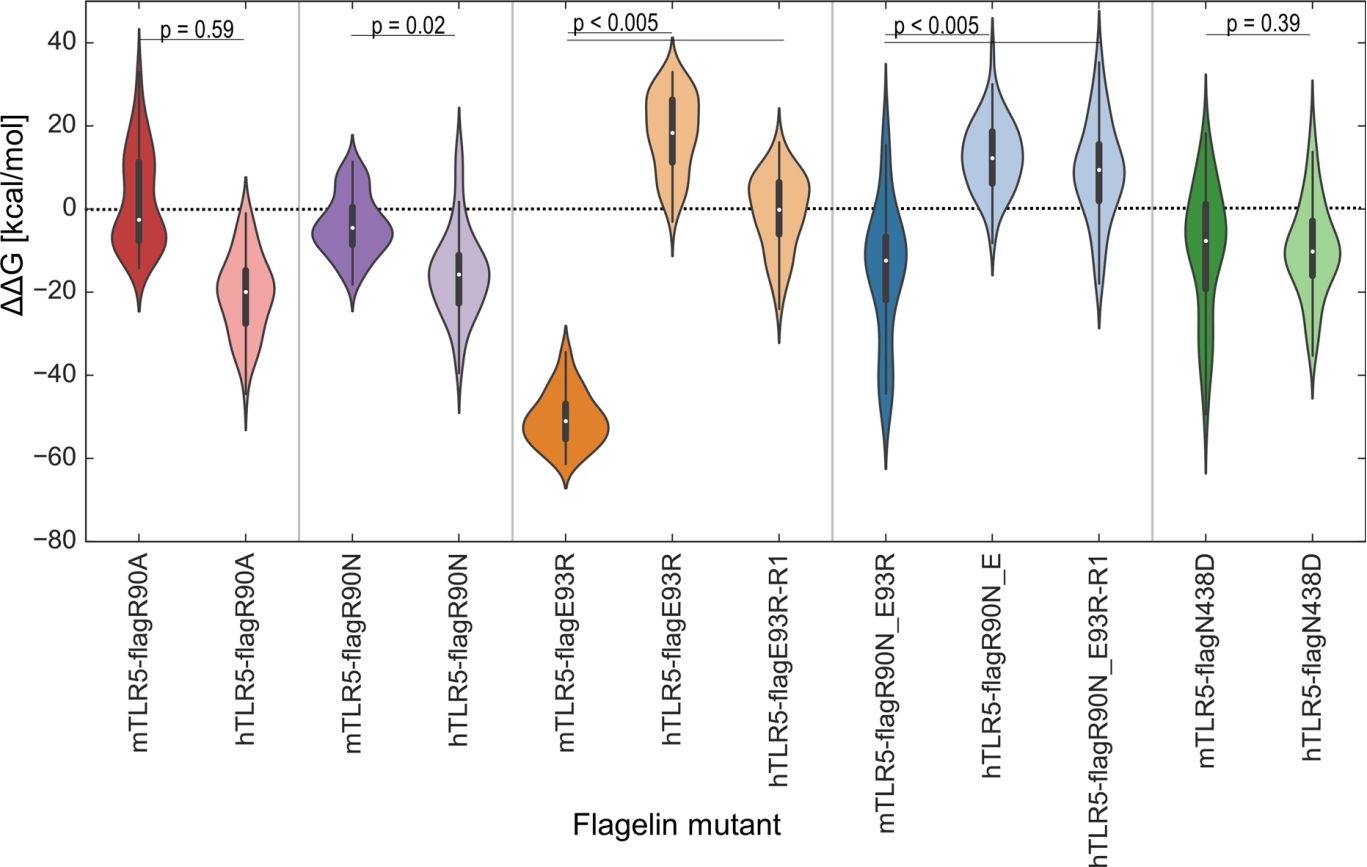
Differences in calculated binding free energies between wild-type and mutant flagellin. The free energy of binding was estimated using MM-GBSA. The energy differences between wild-type and mutant (ΔΔG) flagellins were averaged over 1 ns intervals to obtain 60 points and shown as violin plots. A higher value corresponds to lower stability. The results agree well with our experimental data. The R1 suffix designates an alternative starting rotamer of E93R as described in the methods section. (***p > 0.005, two-tailed unpaired Student’s t-test).

Wild-type SaTy flagellin and a double mutant of flagellin (R90N-E93R) were simulated with hTLR5 and mTLR5 in explicit water (**Fig 4**and **Fig 5**). After 60 ns, the interface between hTLR5 and wild-type flagellin (**Figs 4A and 5A**) and that between mTLR5 and wild-type flagellin (**Figs 4B and 5B**) remained stable and similar to the initial homology structure. The interface between mTLR5 and the mutant flagellin, too, remained stable (**Figs 4D, 5E and 5F**). In contrast, the interface between hTLR5 and the mutant flagellin underwent some structural changes (**Fig 4C, Fig 5C and 5D**), specifically, in the LRR9 loop (shown as a thick blue band) within the primary binding site of hTLR5 dissociated from flagellin. The flagellin D1 fragment in this complex assumed a different binding position across the primary and secondary interfaces. The results of the simulation agree very well with experimental results, in which this specific flagellin double mutant fails to activate hTLR5 but activates mTLR5 (**Fig 1C**). The binding energies of flagellin and mutant flagellin to h- and mTLR5 were estimated from the simulation and were found to correlate well with the experimentally determined receptor activation (**Fig 6**). For mutants R90A, R90N and N438D, the estimated ΔΔG for hTLR5 was comparable to that of mTLR5 (**Fig 6**). The differences of estimated ΔΔG’s for the E93R mutants binding to h- and mTLR5 were similar to the double R90N-E93R mutant suggesting that the main contribution to observed changes in the ΔΔG for the double R90N-E93R mutant originates from the E93R mutation, while experimental results show that this mutation does not decrease receptor activation (**Fig 1B**).

**Fig 7:**
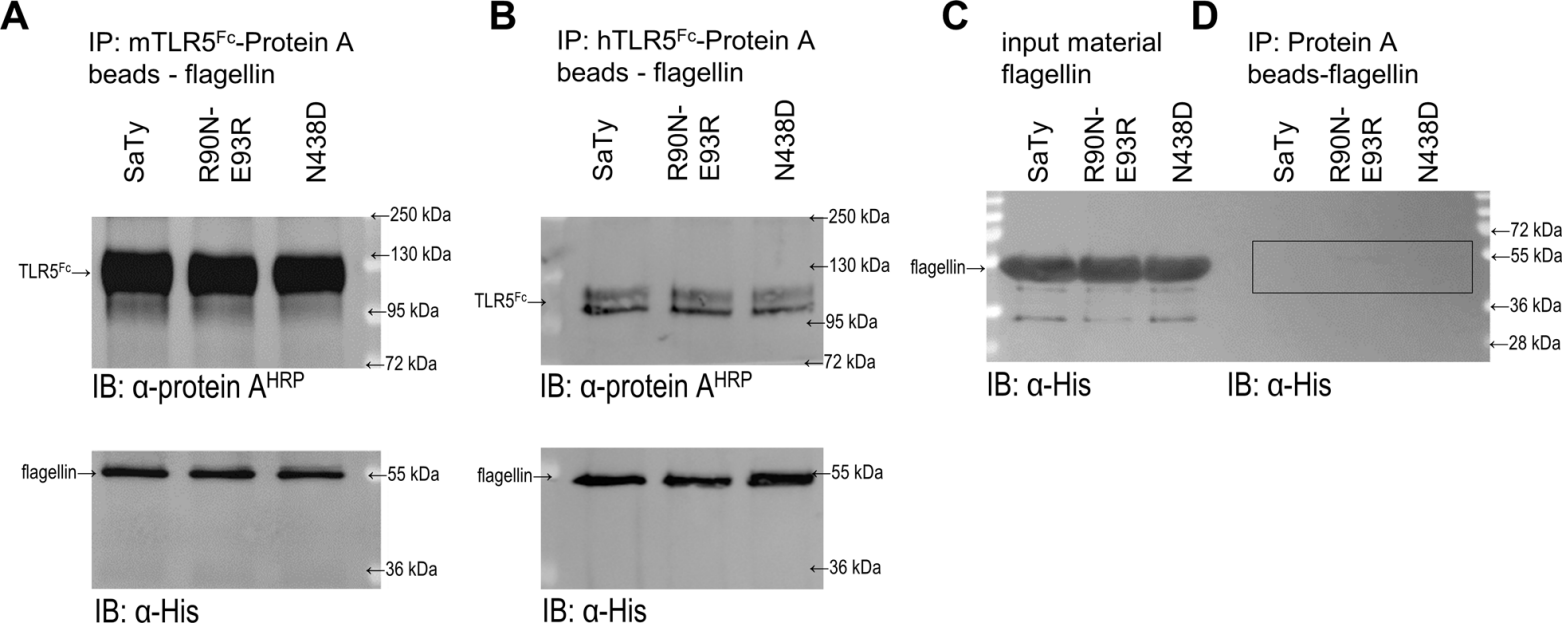
Mutants bind to TLR5 as efficiently as wild type flagellin. (**A**,**B**) The ectodomain mTLR5-Fc (mTLR5^Fc^) (**A**) or hTLR5-Fc (hTLR5^Fc^) (**B**) was immobilized on Protein D beads and mixed with equal amounts of purified wild-type flagellin or mutants. Bound flagellins and TLR5 were eluted and detected by Western blot using Protein-A conjugated with HRP for TLR5 (top panel) and anti-His antibodies for flagellins (bottom panel). (*Data are representative of 2 independent experiments*.) (**C**) Quality of input material. (**D**) Unspecific binding of flagellins to Protein-D beads.

Immunoprecipitation studies show that binding of the R90N-E93R and N438D mutants to h- and mTLR5 was as efficient as binding of wild-type SaTy flagellin (**Fig 7**). This might seem surprising yet was not altogether unexpected since a previous study has also shown only slightly weaker binding of the completely inactive mutant R90A to hTLR5 compared to wild type flagellin [7]. Furthermore, a total of three amino acid residues (Q89A/R90A/Q97A) had to be altered in the *S*. *dublin* flagellin to decrease binding to *dr*TLR5 [24]. Therefore, single amino acid changes which attribute to a decreased activation level do not necessarily also affect binding, since extensive ligand surfaces are involved in the multiple interaction interfaces. The effects of the single point mutations might rather be due to insufficient receptor conformational changes decreasing efficient dimer formation.

## Discussion

Species-specific ligand recognition has been reported for a number of receptors in the TLR family, including TLR4 [4,41], TLR9 [2,3], TLR2 [5], and TLR5 [6–8]. In this study, we focused on the species-specific differences in recognition of flagellin between hTLR5 and mTLR5. The primary binding site between flagellin and TLR5 has been described in the crystal structure of the complex of a fragment of *dr*TLR5 with truncated flagellin of *S*. *dublin* (containing the D1 and D2 domains) [24]. The crystal structure of the *dr*TLR5 fragment provides the basis for interpreting the results of the mutational analysis performed on human and mouse receptors.

To apply the structural information of *dr*TLR5 to the mode of recognition of flagellin by hTLR5 and mTLR5, and to explain differences in recognition between the two species, we constructed homology models of hTLR5 and mTLR5 in complex with *S*. *typhimurium* flagellin based on the crystal structure of the *dr*TLR5–*S*. *dublin* complex. Data pertaining to the crystal structure indicates that the amino acid arginine at position R90 in flagellin and several other surrounding amino acids form a so-called ‘hot spot’ of the primary binding interface between flagellin and TLR5 [24]. All amino acid residues selected in this study are located within the highly conserved block of 140 residues of flagellin that corresponds to the ND0, ND1a and ND1b sub-domains and the β-turn. A study by Beatson et al. aligning 20 of the most diverse flagellin sequences shows that residues at positions 83, 90 and 93 are consistently of a charged or polar nature, residues at position 430 and 431 are consistently polar even among unrelated species and residues at positions 438 and 442 are more variable among species (numbering is relative to *Salmonella typhimurium* flagellin) (**Fig 1A**) [22]. The

R90D mutation completely abrogates hTLR5 and significantly decreases mTLR5 activation (**Fig 1B**). Mutational studies agree with the data from our model, which shows a similar orientation of mouse and human amino acid side chains corresponding to the zebrafish counterparts that interact with flagellin residue R90 in the crystal structure. Conservation of this amino acid position among distinct bacterial species is quite high, and the mutation R90D renders bacteria completely immobile, while mutating arginine 90 to alanine or asparagine has a somewhat milder effect on motility and on the activation of the murine receptor, as reported in previous studies [7]. In contrast, hTLR5 exhibits high sensitivity to mutations at this flagellin position.

Several flagellin point mutations of amino acid residues, which come into contact with *dr*TLR5 in the crystal, resulted in abrogated activation of hTLR5 but not of mTLR5. Residue R431 corresponds to R441 in *S*. *dublin*, and it interacts with L56 and Q90 in *dr*TLR5. Although the amino acid residues are similar in this area between the mouse and the human receptors, the flagellin point mutation seems to exploit a diverse effect in activation, with a minor effect on the mouse receptor and a relatively high effect on the human receptor. The same holds true for flagellin residue N438 (corresponding to *S*. *dublin* N448). Again, the mouse receptor seems to be only slightly affected by a mutation to aspartic acid, while activation of the human receptor is reduced by nearly 70%. The predicted binding energies (**Fig 6**) and the experimental results of binding (**Fig 7**) of the flagellin mutant N438D to h or mTLR do not show any differences compared to wild type flagellin. A single point mutation in this region is clearly not enough to decrease binding, but does impact receptor activation. Since the mutation is located within the primary binding site, a direct impact on receptor dimerization is not expected, although an indirect impact through receptor conformational changes cannot be excluded.

Mutagenesis of other investigated residues of flagellin resulted in more subtle effects even on hTLR5, indicating that their participation in binding with TLR5 is minor and more than one point mutation needs to be introduced to abolish activation, as can be seen for the double mutant R90N-E93R, whose effect is synergistic (**Fig 1B**).

Flagellin residue E93 seems to be quite crucial in the primary binding interface with *dr*TLR5 because it interacts with four residues of *dr*TLR5 (N213, K242, N268, and F278) [24]; however, the mutation of flagellin E93 to arginine decreased neither hTLR5 nor mTLR5 activation (**Fig 1B**), although the predicted binding affinities were different between hTLR5 and mTLR5 (**Fig 6**).

An interesting issue arises when analyzing results of activation and binding of mutations R90N and E93R in a single or double variant to hTLR5. A simultaneous mutation of both residues has a profound synergistic effect on the activation potential of hTLR5 and shows a decrease in binding efficiency (**Fig 1B**). Calculated ΔΔG values of single and double mutants suggest that binding could be in a larger part attributed to residue R93 while experimental results of double and single mutants suggest activation to be attributed in a larger extent to residue R90, although a combination exerts a synergistic effect. Pull down experiments, on the other hand, show comparable binding of all mutants and wt flagellin to TLR5 (**Fig 7**). Upon flagellin binding, the LRR9 loop of TLR5 undergoes a conformational change from a flexible structure to a rigid groove into which R90 is deeply inserted. Neighboring flagellin residues in the groove (including E93) are responsible for half of the H bonds in the primary interface and form a relatively large buried surface area (35% of the primary interface) [24]. Insertion of R90 could be a major precondition for receptor confirmation changes of the LRR9 loop enabling receptor dimerization (the precondition for activation), and while a single mutation at this position is insufficient for abolishing primary binding of the ligand, it could be enough to abolish signaling to a certain extent through influencing these structural rearrangements. In agreement with this theory, three amino acid residues within the primary interface had to be changed to affect binding in the crystal structure study [24], while a single mutant (R90A) abolished hTLR5 activation although its binding was also detected by immunoprecipitation in a different study [7]. While a single mutation in the primary binding site would alter binding of the analyzed fragment, numerous interactions between ligand and receptor throughout the whole flagellin molecule overrun the effect. A double mutation on the other hand intensifies the moderate detrimental effects of both single mutations.

Comparison of the analyzed regions between h- and mTLR5 suggest similar binding interfaces and no obvious significant differences were noticed between mTLR5 and hTLR5. Nonetheless, the mutagenesis results indicate more robust activation of mTLR5 in comparison to the human homologue. mTLR5 was nearly insensitive to single- and double-point mutations in the primary binding area analyzed in this study (**Fig 1B, Fig 2**).

The reasons for different activations of hTLR5 and mTLR5 are difficult to pinpoint in terms of a single amino acid residue. It is very encouraging that the all-atom simulations captured the subtle effects of combined amino acid differences between h- and mTLR5 for the R90N-E93R double flagellin mutant. The largest structural differences were observed in the LRR9 loop (**Fig 4C**), where a histidine in hTLR5 is replaced with a glutamine in mTLR5 (and an alanine with proline) [24]. The LRR9 loop is in contact with residues 90 and 93 in flagellin and was dissociated from the flagellin double R90N-E93R mutant in hTLR5 but not in mTLR5 which is in agreement with experimental results of activation (**Fig 1B**).

More potent recognition and binding of other regions of flagellin, which is also crucial for receptor activation by the mTLR5 receptor, could compensate for the effects of mutations in the primary binding region and, therefore, promote insensitivity to the analyzed single point mutations. However, these issues exceed the scope of the present study. Considering the higher and more diverse commensal burden of mice compared to humans, the evolutionary scope of the species-specific responses noted in this study could be a consequence of the differential requirements of rodent and human innate immunity. Previous studies have, in fact, already noticed a more potent response of mTLR5 compared to the human counterpart to flagellins of different bacterial species [7], yet the location of this difference has not been established yet.

Flagellin is a remarkable example of evolutionary restriction and tolerance for random fluctuation in molecular evolution with regard to structure and function within a single molecule. The packing of monomeric flagellin into flagellar filaments through intermolecular contacts to the D1 and D0 domains of the neighboring monomers strictly restricts to even minimal changes in these regions. This is reflected in the remarkable conservation between bacterial species, while accentuated differences in the sequences and lengths of the variable domains point out the negligible structural role of this region. Functional flagellae represent a selective advantage for bacteria to find the environmental niche, including host tissues for pathogenic bacteria.

We showed that the ability of mutated flagellin to activate TLR5 is not directly linked to the formation of functional flagella necessary for bacterial motility. The E93R, N430D-N433D, and N430E-N433E flagellins activated TLR5 even when mutations disrupted bacterial motility (**Fig 3**and **Fig 1B**). Only the R90D and, to some extent, the R431D mutations affected the TLR5 activation capacity of flagellin and bacterial motility. Residues at these positions are highly conserved even among unrelated bacterial species and are most likely crucial in the intersubunit connections within the filaments. Mutation E83R is the only one that is very highly conserved among different species but has a surprisingly low effect on motility. While there does not seem to be a simple direct correlation between TLR5 activation and effects on motility, the large majority of tested mutations had some effect on bacterial motility, thus confirming the stringency of structural requirements in this region. However, notably, all tested motile variants activated TLR5 as well.

The species-specific differences presented in this study provide new insights into the understanding of TLR5 activation and function, and they should be considered in drug design, where host and pathogen species should not be overlooked, particularly when designing vaccines adjuvanted by flagellin segments. In other words, it is crucial to work with an appropriate pathogen strain and host when developing vaccines and vaccine adjuvants, and to take into account differential host responses.

## Supporting Information

S1 Fig Root mean square deviation vs. simulation time. (TIF)

S2 Fig Root mean square deviation of TLR5 LLR9 loop vs. simulation time. (TIF)

S3 Fig Dictionary of protein secondary structure (DSSP) of TLR5 LLR9 loop vs. simulation time. (TIF)

S4 Fig ERRAT confidence limits for mTLR5-flagellin complex before and after the MD simulation. (TIF)

S5 Fig ERRAT confidence limits for mTLR5-flagellin complex before and after the MD simulation. (TIF)

S6 Fig Normalized DOPE Z-score vs. simulation time. (TIF)

S1 Table Primers used for constructing chimeric proteins and mutants. (PDF)
