# Supplementary figures and images for: Distinctive Recognition of Flagellin by Human and Mouse Toll-Like Receptor 5
Source: PLoS One. 2016 Jul 8;11(7):e0158894. doi: 10.1371/journal.pone.0158894 (PMC4938411; doi:10.1371/journal.pone.0158894)

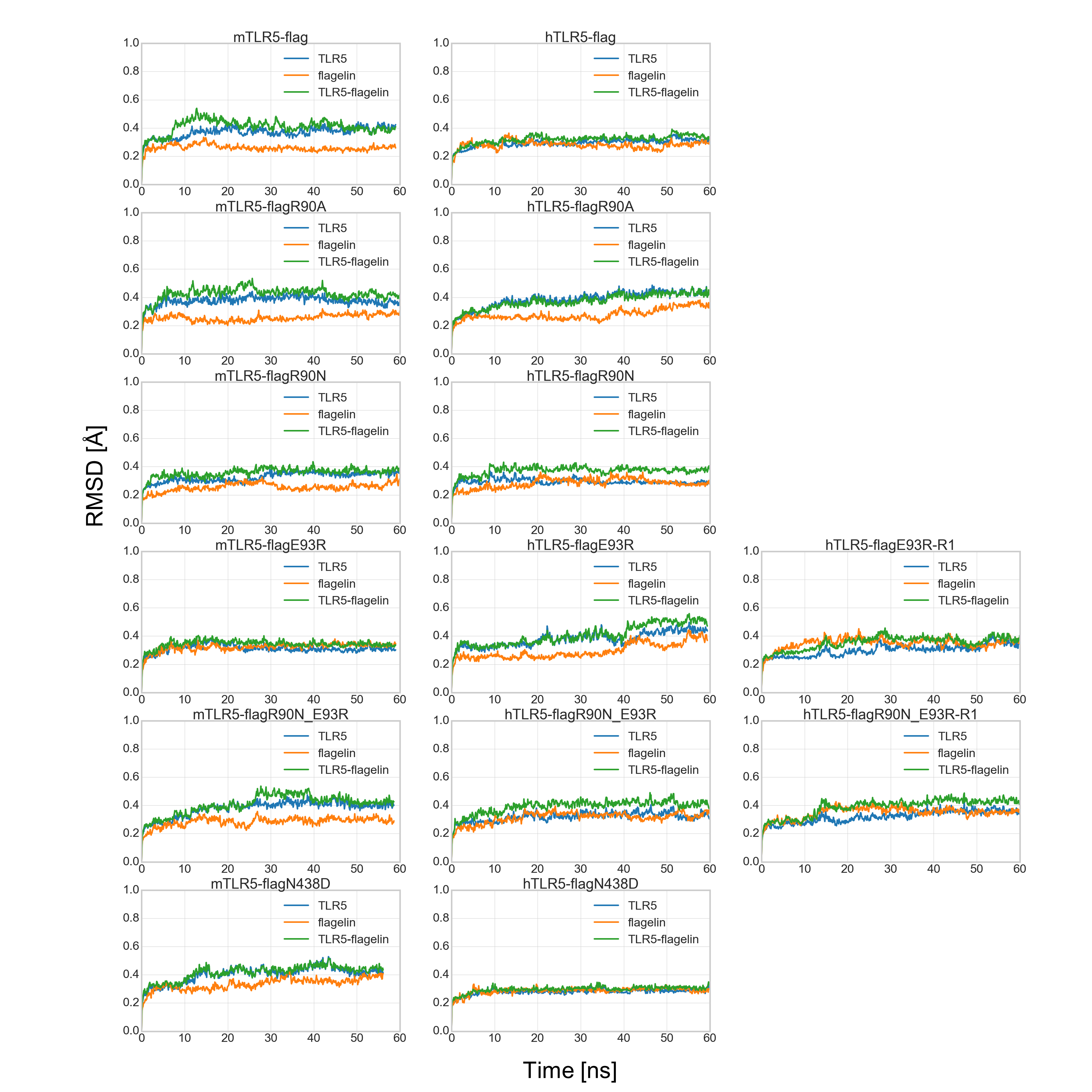

Supplement: S1 Fig — (TIF) [file pone.0158894.s001.tif]

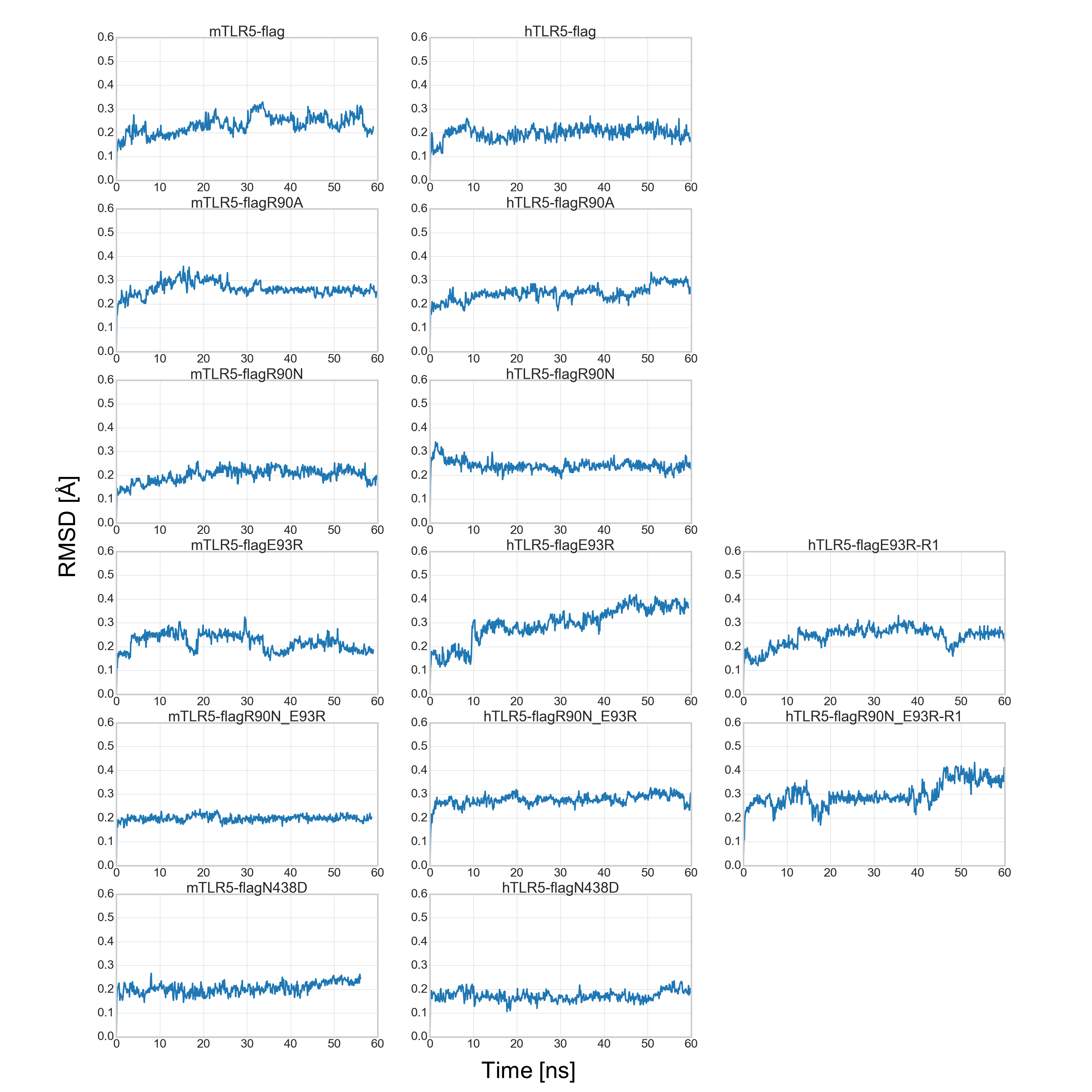

Supplement: S2 Fig — (TIF) [file pone.0158894.s002.tif]

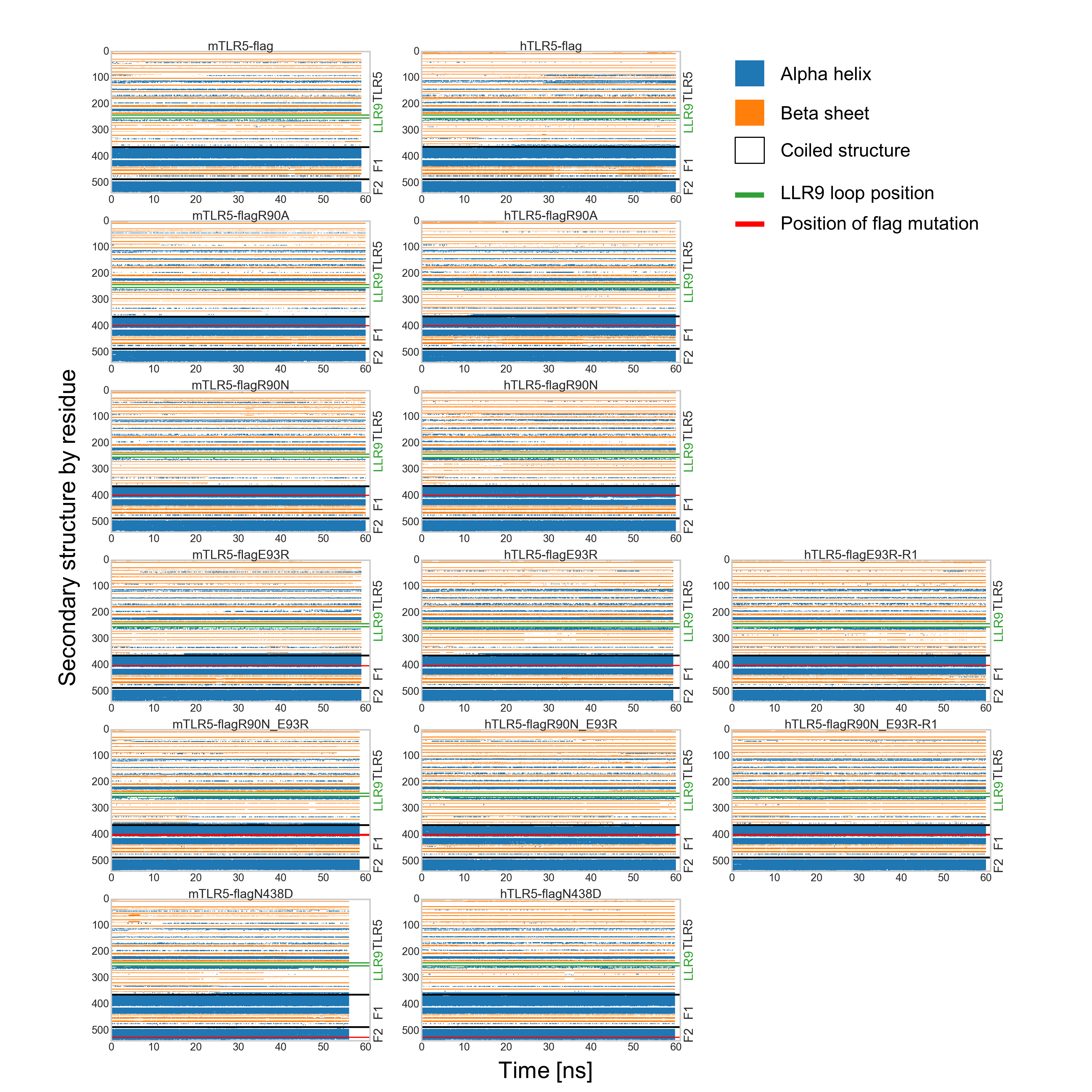

Supplement: S3 Fig — (TIF) [file pone.0158894.s003.tif]

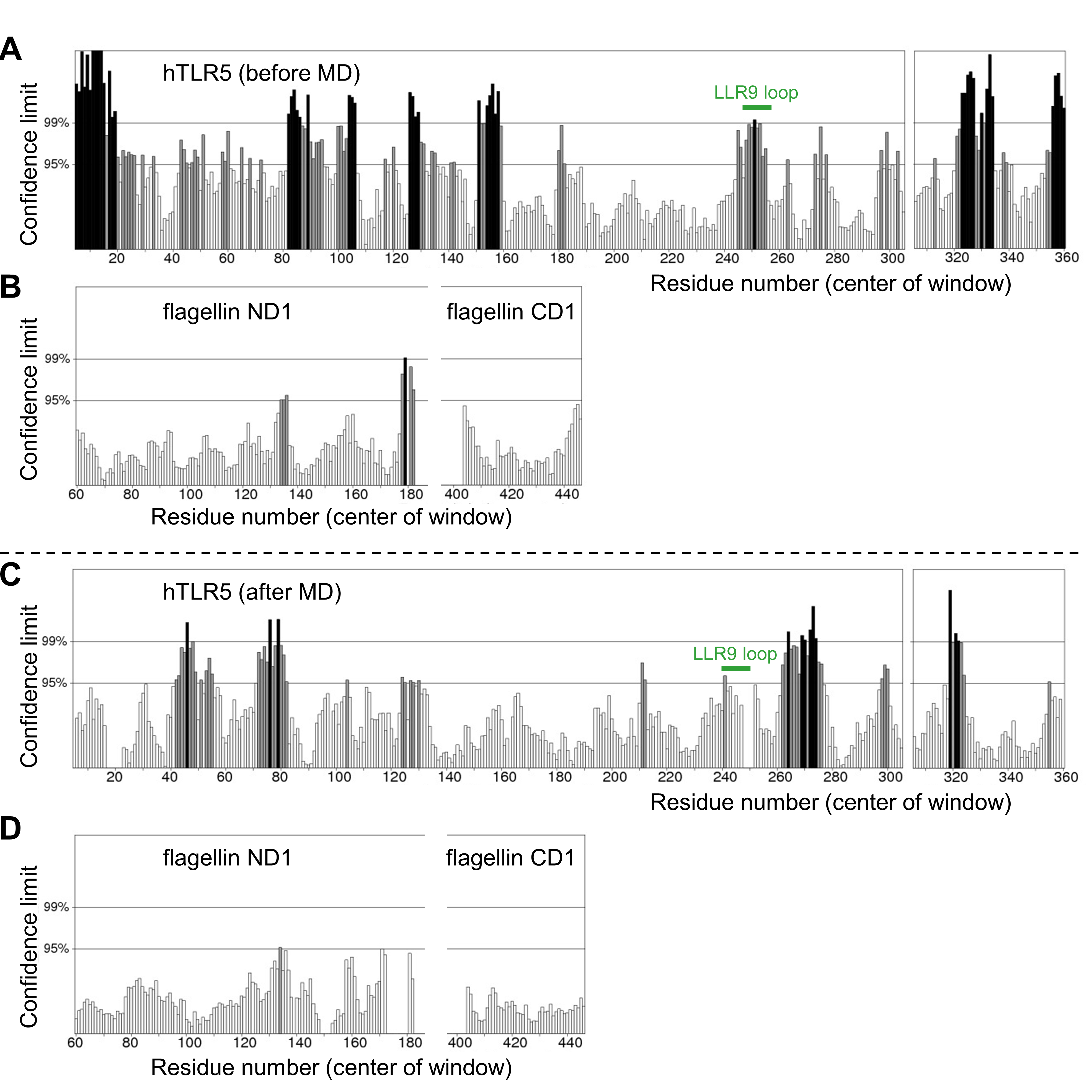

Supplement: S4 Fig — (TIF) [file pone.0158894.s004.tif]

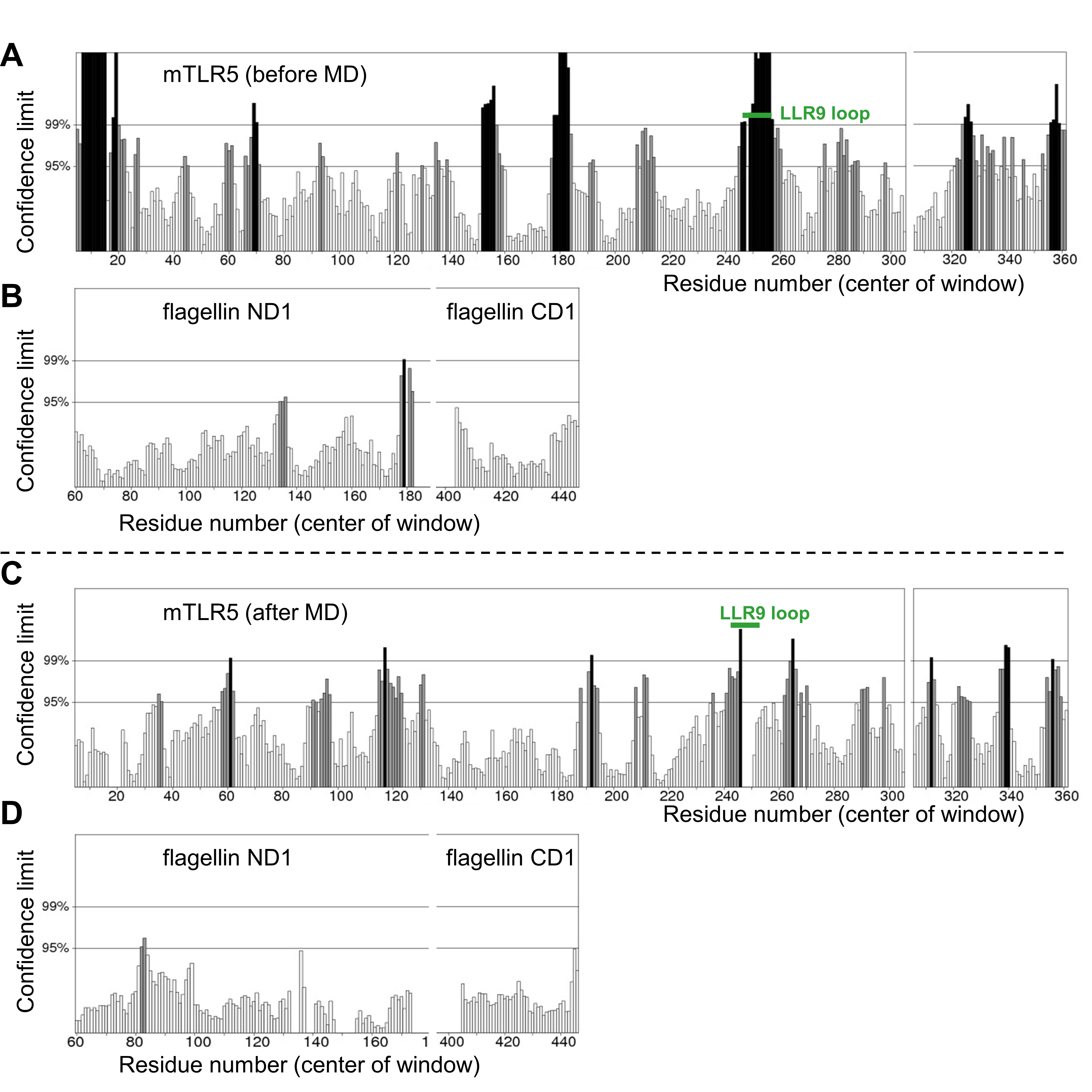

Supplement: S5 Fig — (TIF) [file pone.0158894.s005.tif]

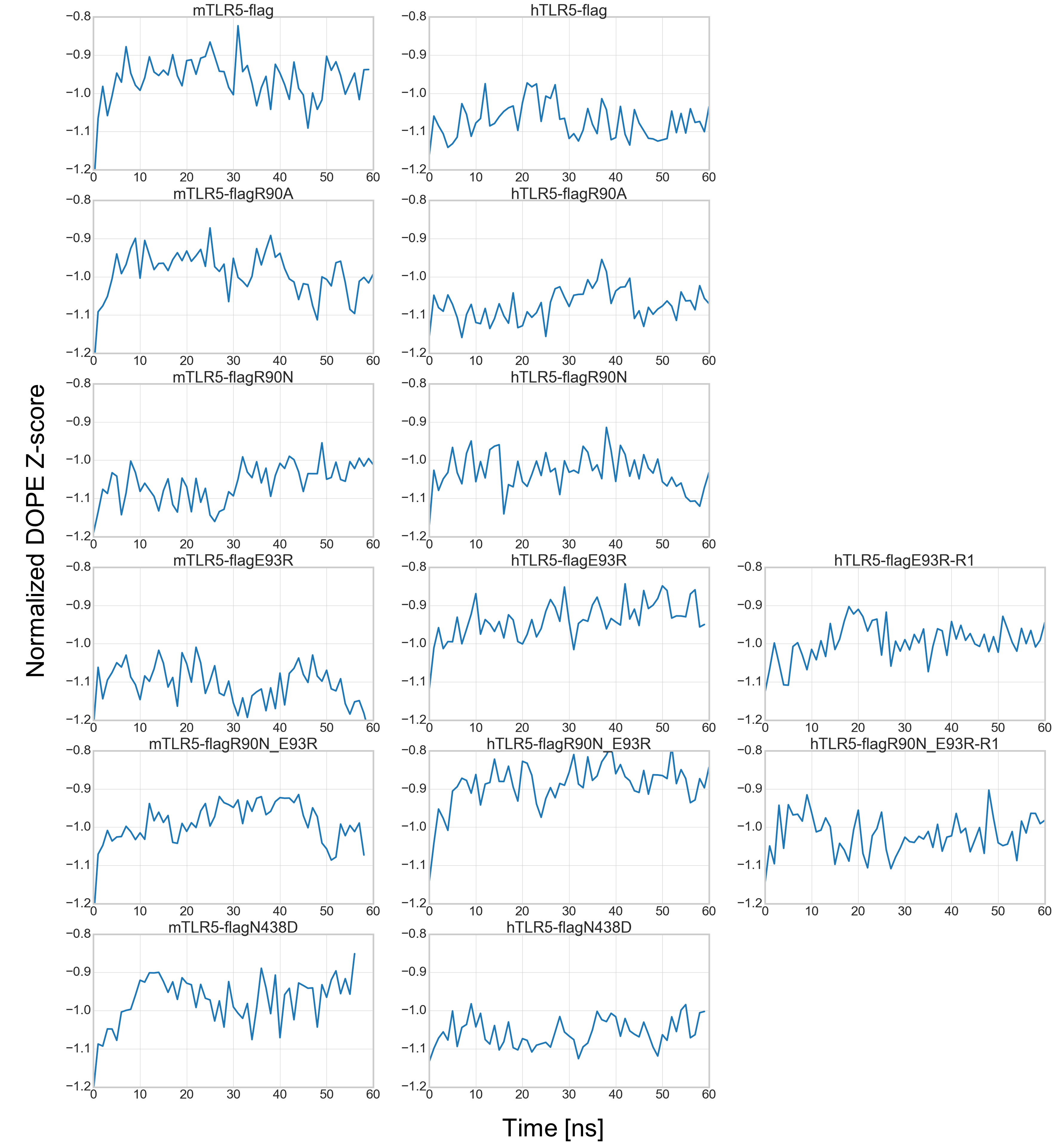

Supplement: S6 Fig — (TIF) [file pone.0158894.s006.tif]
