# Supplementary material for: Distinctive Recognition of Flagellin by Human and Mouse Toll-Like Receptor 5
Source: PLoS One. 2016 Jul 8;11(7):e0158894. doi: 10.1371/journal.pone.0158894 (PMC4938411; doi:10.1371/journal.pone.0158894)
Supplement: S1 Table — (PDF) [file pone.0158894.s007.pdf]

S1 Table. Primers used for constructing chimeric proteins and mutants.

Oligo. no.	primer sequence 5'->3'	Comments	
1-F	CCGCCTCGAGATGGCACAAGTCATTAATACAAAC	SaTy	SaTy and SeMa flagellin were PCR-amplified using DNA isolated from bacteria. The purified PCR products were cloned into the *Xho*I and *Bam*HI sites of the pET19b expression vector containing an N-terminal 6x HIS-tag.
1-R	CCGCGGATCCTTAACGCAGTAAAGAGAGGAC		
2-F	CCGCCTCGAGATGGCACAAGTAATCAACACTAAC	SeMa	
2-R	CCGCGGATCCTTAACGCAGCAGGGACAGAAC		
**SaTy mutations***			
3-F	GAAGGCGCGCTGAACCGGATCAACAACAACCTG	E83R	
4-F	CAACAACCTGCAGGCCGTGCGTGAACTGG	R90A	
5-F	CAACAACCTGCAGGATGTGCGTGAACTGG	R90D	
6-F	CAACAACCTGCAGAACGTGCGTGAACTGG	R90N	
7-F	CAGCGTGTGCGTCGCCTGGCGGTTCAGTC	E93R	
8-F	GTGCGGTACAGAACGATTTCAACTCCGCTATTACC	R431D	
9-F	CTCCGCTATTACCGACCTGGGCAACACCG	N438D	
10-F	CCAACCTGGGCAATCGCGTAAACAACCTGAC	T442R	
11-F	CCAACCTGGGCAACGAAGTAAACAACCTGAC	T442E	
12-F	CGAAATCAACAACAACCTGCAGAACGTGCGTCGCCTGGCGGTTCAGTCTGCTAAC	R90N-E93R	
13-F	CTGACCTGGGTGCGGTACAGGATCGTTTCGATTCCGCTATTACCAACCTGGG	N430D- N433D	
14-F	CTGACCTGGGTGCGGTACAGGAACGTTTCGAATCCGCTATTACCAACCTGGG	N430E- N433E	
Other			
15-F	AGATGCGAGCCACCATGGGAGACCACCTGGACCTT	AU1-hTLR5 (signal sequence as template)	The AU1 tag was inserted between the hTLR5 signal sequence (aa 1-20) and the sequence coding for a mature hTLR5 receptor (aa 21-858) in the pUNO vector. A PCR overlap extension technique was used with pUNO-hTLR5 as the template. The nucleotide sequence of the AU1 tag was incorporated into the 15-R and 16-F primers. PCR product was cloned into *Nco*I and *Xba*I sites of pUNO-hTLR5.
15-R	AAGGAATGATGTAGCGGTATGTGTCTCCAAACACAGGACCGGCCA		
16-F	GTTTGGAGACACATACCGCTACATCATTCCTTCCTGCTCCTTTGA	AU1-hTLR5 (mature TLR5 as template)	
16-R	GCGCGCCGCGCTCTAGATTTTCTAGCCTGTTTTCTGA		
17-F*	CGCCCATTCTCCGCCCGATGGCTGACTAATTTT	Deletion of *Nco*I in pUNO vector	For this purpose, the second *Nco*I restriction site on pUNO-hTLR5 vector was deleted via introduced point mutation.
**Motility test**			
46-F	GTAATTGATAAGGAAAAGATCATGGCACAAGTCATTAATACAAAC	Flagellins as template	Wild-type flagellin and mutants were cloned into the pRP4 vector using the Gibson assembly method [28]. 5 μl of PCR-amplified and -purified DNA fragments, joined in equimolar amounts, was added to 15 μl of the Gibson assembly master mix and incubated for 1 h at 50°C.
46-R	GGTGAATCAATCGCCGGATTAACGCAGTAAAGAGAGGACG		
47-F	GTCCTCTCTTTACTGCGTTAATCCGGCGATTGATTCACCG	pRP4 vector as template	
47-R	GTATTAATGACTTGTGCCATGATCTTTTCCTTATCAATTACAAC		

Legend: Restriction sites, mutation, tag, annealing; * only forward oligonucleotide is given
